# Dynamic Recrystallization Simulation of PH13-8Mo Stainless Steel by Cellular Automata Method Based on Laasraoui–Jonas Dislocation Density Model

**DOI:** 10.3390/ma17235752

**Published:** 2024-11-25

**Authors:** Linli Hu, Shaoshuai Zhou, Xunyu Yuan, Xiwen Xu, Ruizhi Yang, Xiaoyong Shu

**Affiliations:** 1 School of Mechanical Engineering, Jiangxi Vocational College of Mechanical and Electrical Technology, Nanchang 330013, China; 2 Jiangxi Provincial Engineering Research Center for Surface Technology of Aeronautical Materials, Nanchang Hangkong University, Nanchang 330063, China; yyscaicaizi@163.com (S.Z.);

**Keywords:** PH13-8Mo stainless steel, L–J dislocation density model, metacellular automata (CA) model, dynamic recrystallization

## Abstract

The Gleeble-1500 hot simulation experimental equipment was used to investigate the effects of hot simulation compression on PH13-8Mo stainless steel with strain rates ranging from 0.1 to 10 s^−1^ and deformation temperatures ranging from 900 to 1150 °C. The stress–strain charts for each deformation condition clearly show the characteristics of dynamic recrystallization behavior. The rheological stress rises as the deformation temperature falls and the strain rate rises. A coupled Laasraoui–Jonas (L–J) model was developed based on the discovery that the dislocation density is crucial to the nucleation and micro-structure evolution of dynamic recrystallization during thermal deformation. The findings demonstrate that the model accurately captures the combined action of dynamic reversion and recrystallization inside the material during hot compression of PH13-8Mo stainless steel and that as deformation rises, the dislocation density first increases and subsequently drops. For calculations and numerical simulations of the evolution of the micro-structure under hot compression, the created model was integrated into the DEFORM-3D finite element simulation program. The projected micro-structure evolution of the PH13-8Mo stainless steel under various deformation circumstances is compared to the measured grain distribution, grain size, and the degree of dynamic recrystallization in the metallographic pictures. The fact that the simulated result plots resemble the metallographic charts so closely demonstrates that the L–J dislocation density model can reliably forecast how dynamic recrystallization of PH13-8Mo stainless steel would behave under hot compression.

## 1. Introduction

The hot deformation process is critical in improving the overall properties of forgings, with recrystallization playing a key role in reconstructing the alloy’s micro-structure during deformation. By refining the grains and reducing the metal’s deformation resistance, dynamic recrystallization improves the final mechanical properties of the material. Furthermore, it counteracts the work hardening that occurs during thermomechanical treatment. The primary driving force behind this dynamic recrystallization is the dissipation and rearrangement of the dislocation density during thermomechanical treatment [1]. Understanding the dynamic recrystallization process of alloys can help gain fundamental insights, as described by Shu et al. [2] and Yang et al. [3]. The martensitic stainless steel PH13-8Mo undergoes precipitation hardening and has exceptional qualities, including high strength, high toughness, and great corrosion resistance [4,5,6,7,8,9,10]. Although thermomechanical treatments, like forging of PH13-8Mo, are commonly used in the industry and the various deformation and recrystallization processes of PH13-8Mo are important, relatively little is known about the dynamic recrystallization during the forging process as it confers a poor plastic deformation ability and a narrow hot working process window [9,10,11,12,13]. However, with the recent development of fast in situ electronic microscopy, in situ observation of dynamic recrystallization has become feasible [14,15,16,17,18,19]. In the research of some scholars, it has been explicitly stated that there is an urgent need for simulation to predict such experiments. It is crucial to predict or control the microstructural evolution process during the dynamic recrystallization of materials [14,15,16,17,18]. Traditionally, the Cellular Automata (CA) method, with its high efficiency, strong flexibility, and real-time capabilities, has emerged as a particularly effective tool for simulating micro-structure evolution under different strain conditions during the thermomechanical treatment process.

In this work, we aim to build a Laasraoui–Jonas (L–J) dislocation density model of PH13-8Mo stainless steel using the dynamic recrystallization of grains under various compression deformation conditions. Using the micro-structure module of DEFORM-3D finite element simulation software, the micro-structure evolution during thermal deformation was stimulated, and the experimental results of the level of gain sizes and the recyclization degree were compared. The finite element simulation with L–J’s model shows good agreement with the experimental observations. The ultimate goal is to provide theoretical and technical support for the PH13-8Mo stainless steel forging process optimization.

## 2. Materials and Experiments

**Figure 1:**
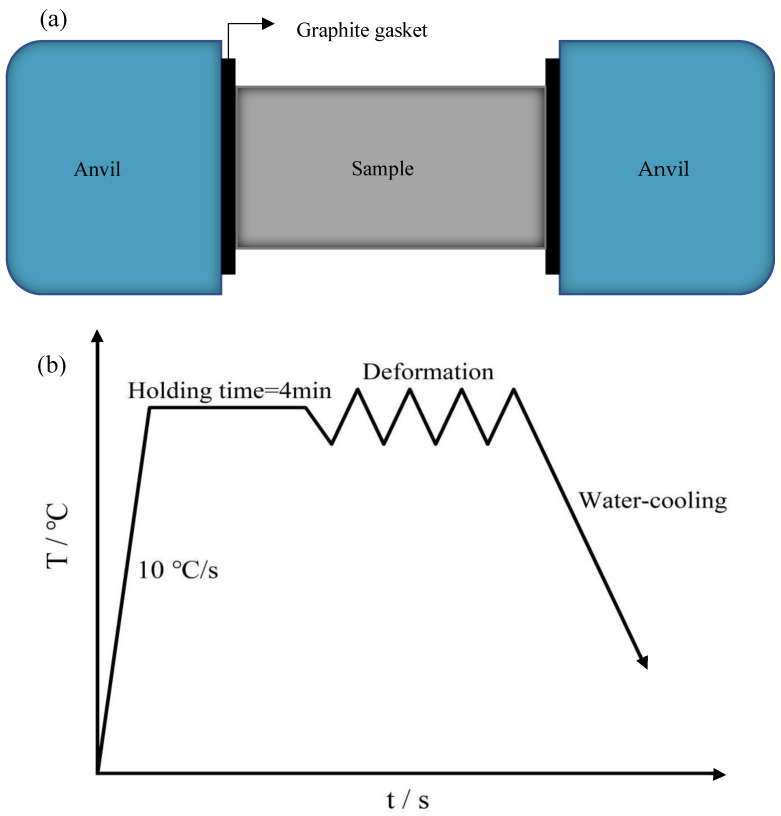
(**a**) Schematic diagram of Gleeble-1500 hot compression test; (**b**) Thermal compression process curve.

The samples were made from annealed PH13-8Mo stainless steel bar, whose chemical composition is displayed in Table 1. Stainless steel bars with PH13-8Mo were processed into an Φ8 mm × 12 mm cylindrical sample by Wire cut Electrical Discharge Machining. A Gleeble-1500 thermomechanical simulation test equipment was used to conduct the thermomechanical simulation compression test, as shown in Figure 1a. At both ends of the specimen, graphite spacers were added to reduce friction between the sample and the tools and therefore prevent the sample from bulging during hot compression. The specimens were resistance heated to the experiment’s required temperature; the specimens were heated to the experimental temperature with a rate of 10 °C/s. After holding the temperature for 4 min, the samples were compressed at temperatures between 900 and 1150 °C with a strain rate between 0.1 and 10 s^−1^; the deformation amount was 50% of the total height of the material, and the objects were cooled with water to mimic a hot working environment. To ensure the accuracy of the experiment, we conducted three experiments on each set of experimental samples. Figure 1b displays the hot compression process graph. The original data from the compression test were used to create the true stress–true strain curve.

The micro-structure was examined both before and after deformation using an optical microscope (COIC, Chongqing, China). KMnO_4_ + H_2_SO_4_ + H_2_O mixed solution was used as the metallographic etching agent to prepare the specimens after thermomechanical treatment for micro-structure imaging. The truncation technique was used to determine the average grain size, and ImageJ (https://imagej.net/ij/, accessed on 7 September 2024) was used to determine the volume fraction of dynamic recrystallization.

The 3D geometry of the forgings, upper die head, and bottom die cutting plate were modeled using commercial finite element modeling software (DEFORM-3D) (https://www.deform.com/products/deform-3d/, accessed on 7 September 2024). In order to validate the accuracy of the finite element model and create a database of deformed materials, the plastic deformation data and grain data were analyzed using the flow stress model and dynamic recrystallization model of PH13-8Mo stainless steel [20,21]. The high-temperature compression process of cylindrical specimens was simulated using commercial finite element modeling software (DEFORM-3D) (https://www.deform.com/products/deform-3d/, accessed on 7 September 2024). in order to validate the model accuracy.

## 3. Results and Discussion

### 3.1. Results of Hot Compression

**Figure 2:**
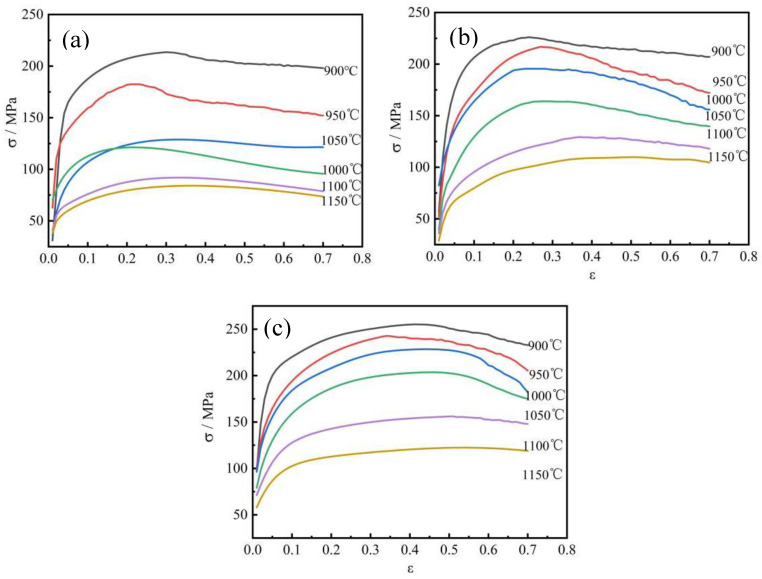
True stress–true strain curves of PH13-8Mo stainless steel under different deformation conditions; (**a**) = 0.1 s^−1^, (**b**) = 1 s^−1^, (**c**) = 10 s^−1^.

The flow curves of PH13-8Mo at strain rates ranging from 0.1 to 10 s^−1^ and deformation temperatures ranging from 900 to 1150 °C are shown in Figure 2. Deformation temperature and strain rate had a substantial impact on flow stress, as shown in Figure 2. The true stress–strain curve of PH13-8Mo steel can be described using the variation law as follows: The true stress rapidly increases in the initial stage of deformation. Subsequently, the rise gradually becomes gentle and the stress decreases, reaching the final stage of deformation. The segment continues to rise, when the strain rate remains constant, corresponding to a specific value. The strain value and deformation resistance decrease with the increase in deformation temperature. When the deformation temperature remains constant, the deformation resistance increases with the increase in strain rate. In the early stages of thermomechanical treatment, a steep rise in the flow stress is followed by a gradual reduction in the strain hardening rate until the peak stress is reached. Finally, a stable state is reached after a gradual reduction in flow stress. This is due to competition between the processes of dynamic softening and hardening during the thermomechanical treatment. At the beginning of deformation, as the strain increased, the density of dislocation multiplied continuously, leading to a large number of entangled dislocations and causing grain elongation, fragmentation, and fibrosis. This phenomenon is known as process hardening, and as the strain increases, the dominant rheological stress rises quickly until it reaches the peak flow stress. The dislocations vanish and reorganize as the duration of high-temperature deformation lengthens and austenite experiences dynamic reversion, polygonization, and dynamic recrystallization. At this point, the rheological stress starts to decrease as the softening effect progressively replaces the work hardening. The rheological stresses achieve a stable stage at the conclusion of deformation, and the softening effect and work hardening reach a dynamic equilibrium.

The flow stress of the PH13-8Mo stainless steel, as shown in Figure 2, decreases with an increase in the deformation temperature at the same strain rate. The increased value of the adjacent curve flow stress at low deformation temperature (900~1050 °C) is higher than that at high deformation temperature (1100 °C, 1150 °C). Because of the stronger thermal activation at high deformation temperatures, the atomic and vacancy diffusions, as well as dislocation cross-slip and climbing, have larger driving forces [1]. The quantity of alloy elements dissolved increases at high deformation temperatures, which favors the occurrence and progression of dynamic recovery and dynamic recrystallization. The PH13-8Mo stainless steel is a positive deformation temperature-insensitive substance, as evidenced by the fact that it experiences less rheological stress at high deformation temperatures. As a result, the PH13-8Mo stainless steel should be chosen for heated working at the proper deformation temperature. At the same temperature, the flow tension and strain rate are proportional. Because the speed of dislocation propagation and the critical shear stress were low, the work-hardening effect was relatively weaker at low strain rates. There was sufficient time for dynamic softening and dislocation removal; hence, the flow stress is lowered. A part of the slip system is also activated, which lowers the material’s resistance to deformation, and the peak stress advances as the strain rate decreases.

### 3.2. The L–J Dislocation Density Model

The dislocation density plays a very important role in the nucleation and tissue evolution of dynamic recrystallization during thermal deformation. The combined effect of work hardening, dynamic recovery, and dynamic recrystallization during thermomechanical treatment is responsible for the change in dislocation density. In this paper, an improved coupled L–J model was used to provide a more realistic description of the evolution of dislocation density during dynamic recrystallization [1,19,21,22], as follows:

(1) $$\frac{d\rho }{d\varepsilon }=h-r\rho -rdV$$

(2) $$h=h_{0}(\frac{\dot{\varepsilon }}{\dot{\varepsilon_{0}}}{)}^{m}exp(\frac{mQ}{RT})$$

(3) $$r=r_{0}(\frac{\dot{\varepsilon }}{\dot{\varepsilon_{0}}}{)}^{-m}exp(-\frac{mQ}{RT})$$

Here R is the general gas constant; Q signifies the diffusion activation energy; T embodies the absolute temperature; $\dot{\varepsilon }$ represents the strain rate; $\dot{\varepsilon_{0}}$ is the strain rate correction constant (generally 1); ρ represents the dislocation density; $h_{0}$ is the hardening index; h is the average strain hardening rate coefficient; $r_{0}$ is the softening constant; r is the dynamic recovery softening coefficient; dV is the volume swept by mobile boundaries; and m characterizes the material strain rate sensitivity exponent.

In the L–J model, the change in dislocation density caused by grain boundary migration is very small and can be ignored. Equation (1) can thus be changed as follows:

(4) $$\frac{d\rho }{d\varepsilon }=h-r\rho$$

Equation (4)’s first-order linear differential equation can be solved, allowing the following conclusions to be drawn:

(5) $$\rho =\frac{h}{r}-\frac{h-\rho_{0}r}{r}exp(-r\varepsilon )$$

In addition, under any condition, the flow stress is proportional to the square root of the dislocation density [23,24]. Thus, it can be inferred that:

(6) $$\sigma =\alpha \mu b\sqrt{\rho }$$

Here α represents the Taylor factor (generally 3.1); and μ and b are the shear modulus and the Burgers vector of the material, respectively.

When the strain is high enough, the function relationship exp(−rε) will approach 0, so the flow stress and recovery stress are almost similar; thus, it can be inferred that:

(7) $$\sigma_{sat}\approx \alpha M\mu b\sqrt{\frac{h}{r}}$$

Substituting Equations (5) and (6) into Equation (7) results in the following expression:

(8) $$\sigma ={[\sigma }_{sat}^{2}-(\sigma_{sat}^{2}-\sigma_{0}^{2})exp(-r\varepsilon ){]}^{0.5}$$

Using the derivation transformation of Equation (8), the following may be demonstrated:

(9) $$\sigma \frac{d\sigma }{d\varepsilon }=0.5r\sigma_{sat}^{2}-0.5r\sigma^{2}$$

Here $\sigma_{0}$ is the yield stress. According to the explanation of the work hardening rate $\theta =\frac{d\sigma }{d\varepsilon }$ [25], Equation (9) can be articulated as follows:

(10) $$\sigma \theta =0.5r\sigma_{sat}^{2}-0.5r\sigma^{2}$$

The softening coefficient r under different deformation circumstances may be obtained using Equation (10). The slope of $\sigma^{2}-\sigma \theta$ (the functional relationship between $\sigma^{2}$ and σθ) can be set as k; then k = −0.5 r, i.e., r = −2 k.

**Figure 3:**
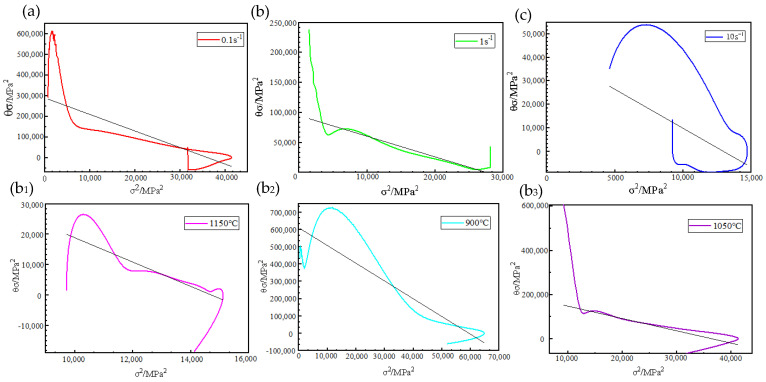
Relationship curves of PH13-8Mo stainless steel during hot compression deformation and curve trend: (**a**–**c**) deformation temperature: 1050 °C; (**b_1_**−**b_3_**) strain rate: 1 s^−1^.

Based on the true stress–true strain data gained from the above hot compression test of PH13-8Mo stainless steel, A $\sigma^{2}-\sigma \theta$ (the functional relationship between $\sigma^{2}$ and σθ) curve relationship graph is established, as shown in Figure 3. The black straight line in the figure represents the approximate trend of the curve generated by the software.

Substituting *ε* = 1 into Equation (3) and taking the logarithm of both sides of Equation (3), one can deduce Equation (11):

(11) $$Inr=Inr_{0}-mIn\dot{\varepsilon }-\frac{mQ}{RT}$$

Substituting the values of r obtained for different deformation conditions into Equation (11) and performing multivariate linear fitting, one can calculate that m = 0.1406, $r_{0}$ = 217.41, and Q = 567,320 J/mol. Substituting this into Equation (3), it can be shown as follows:

(12) $$r=217.41(\dot{\varepsilon }{)}^{-0.1406}exp(-\frac{79765.192}{RT})$$

By extending the work hardening curve θ–*σ* to θ = 0, the dynamic recovery saturation stress may be calculated. Equation (7) may be changed to determine the hardening coefficient, which is h_0_ = 1.12.

## 4. Micro-Structure Simulation and CA Model Validation

### 4.1. Finite Element Modeling

**Figure 4:**
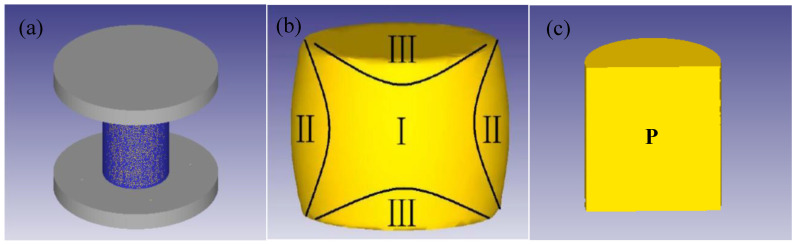
(**a**) Three-dimensional view of the thermal compression model; (**b**) schematic diagram of the deformation area; (**c**) location of the tracking points on the workpiece.

The 3D modeling for the example was performed using commercial finite element modeling software (DEFORM-3D). The measurements of the simulated compression specimens and the experimental specimens were identical, as seen in Figure 4. The element type used in this paper’s modeling of forging was tetrahedron. Grids of varying sizes were used to separate the workpiece, indenter, and cutting plate. The mesh size of the workpiece was refined to 2 mm, and after the mesh division was completed, there were a total of 15,000 elements. This refinement was performed to increase the accuracy of the simulation outcomes on the workpiece. After the meshing was completed, there were 8000 elements overall. The indenter and cutting plate’s mesh size were slightly larger to reduce the calculation volume. The indenter is die steel grade AISID3 [19].

The test workpiece and virtual workpiece both have the same compression temperature. The estimated deformation temperatures were 900 °C, 950 °C, 1000 °C, 1050 °C, 1100 °C, and 1150 °C. The following conditions must be met: a strain rate of 10 s^−1^; contact friction between the workpiece and the tool set to shear friction, with a friction value of 0.3; and model deformation (upsetting ratio) of 50%, or 6.00 mm/h(0) (12) mm/h(6) mm. Given the quickness of the compression procedure between the workpiece and the die as well as between the workpiece and its surroundings, heat convection and radiation had little influence on the temperature change and can be disregarded. Since the hammer forging speed (the indenter’s feed speed) cannot be regulated to have a uniform strain rate, the die feed speed v can only be taken as an average speed [26]. The calculation formula is as follows:

(13) $$v=\dot{\varepsilon }\frac{h_{0}+h}{2}$$

Here h is the height of the workpiece after processing, h_0_ represents the initial height of the workpiece, and v is the mold feed speed.

### 4.2. Numerical Simulation of Dynamic Recrystallization Meta CA

Using the CA technique, which is a discrete grid dynamic model in time, space, and grain state, it is possible to see exactly how the micro-structure changes and develops throughout the dynamic recrystallization [19,26]. Tracking markers (P) are placed on the workpiece in the middle of the significant deformation zone (I) after opening the micro-structure post-processing window. Figure 4b,c displays the tracking spots’ distribution.

### 4.3. Theoretical Reasoning and Models of the CA Method

(1) Boundary conditions and their settings

Cells were typically defined in the setup of the CA method as infinitely expandable in any direction. But in real simulation, it was nearly impossible to achieve an infinite lattice range, so the boundary conditions of the grid must be set. In this simulation, the lateral and vertical boundary conditions were both handled as periodic surround sounds. The flow stress did not affect the grain border conditions, and a Moore-type neighborhood was used. To put it in other words, it was made up of a center cell, four cells along the top and bottom, and four vertex corners close by. The lattice’s overall dimension was equal to the neighborhood’s radius [27,28,29].

(2) Dislocation density model

The dislocation density model used in the CA method is expressed in successive increments as follows:

(14) $$\frac{d\rho_{i}}{d\varepsilon }=h-r\rho_{i}-rdV$$

(15) $$h=h_{0}(\frac{\dot{\varepsilon }}{\dot{\varepsilon_{0}}}{)}^{m}exp(\frac{mQ}{RT})$$

(16) $$r=r_{0}(\frac{\dot{\varepsilon }}{\dot{\varepsilon_{0}}}{)}^{-m}exp(-\frac{mQ}{RT})$$

(17) $$N_{r}=(\frac{(\#Rows)(\#Columus)\sqrt{2}}{K}{)}^{2}h(d\varepsilon {)}^{\left(1-2m\right)}$$

Here $\rho_{i}$ represents the dislocation density of the i-cell; h denotes the work hardening rate; h_0_ is the process hardening constant (the calculated hardening constant h_0_ = 1.12); r denotes the dynamic response rate; r_0_ is the dynamic response constant (the calculated response constant r_0_ = 217.41); $\varepsilon_{0}$ is the strain rate calibration constant (no calibration is required in this case (generally 1)); m is the strain rate sensitivity constant (generally 0.2 for high temperature deformation); Q represents diffusion activation energy (the calculated activation energy Q = 567.32 KJ/mol); Nr denotes the number of randomly selected metacells at each time point; (#Rows)·(#Columns) denotes the number of metacells in a region consisting of several rows and columns; and K is a constant (usually 6030) [30].

(3) Nucleation and growth of dynamic recrystallization

The material’s dislocation density rose with the increase in strain. However, when the strain hit a critical point, the dynamic recrystallization at the grain boundary began to nucleate at a specific rate, consuming the dislocation density as it grows. For the dynamic recrystallization of PH13-8Mo stainless steel, the critical dislocation density was 0.02, and the nucleation rate after criticality was 0.01. The migration of grain boundaries, which occurred during the development process of dynamically recrystallized grains, had a constant migration rate of 1 [31].

(4) Flow stress and material constants

There is an image-only model of the material during work-hardening, and the rheological stress $\sigma_{f}$ of the metallic material during hot deformation can be expressed as follows:

(18) $$\sigma_{f}=\sigma_{0}+\sigma_{p}+a_{1}Gb\sqrt{\rho_{i}}+Gb(\frac{a_{2}}{\delta }+\frac{a_{3}}{D})$$

(19) $$\sigma_{p}=\frac{AGb}{1.24\times 2\pi }[\frac{1}{\lambda }ln(\frac{\lambda }{b})]$$

(20) $$\lambda =0.8[(\frac{\pi }{f_{r}}{)}^{0.5}-2]r_{d}$$

Here $\sigma_{0}$ represents the yield stress; $\sigma_{p}$ represents the peak stress, which can be determined from the rheological stress of the thermal simulation test; b is the Burgers vector (for alloy steel, it is generally taken as 2.45 × 10^−10^ m); G is the shear modulus (for alloy steel, it is generally taken as 8.1 × 10^6^ MP); $a_{1}$ represents the dislocation interaction coefficient (which is taken as 1 for most metallic materials); D epitomizes the average grain size (where the original grain size is taken); δ characterizes the average sub-grain size; $a_{2}$ and $a_{3}$ are the effect coefficients of D and δ on dislocation, respectively (both take the value 0.1); and A is a constant (with a value of 1).

### 4.4. Analysis of the CA Simulation Results

**Figure 5:**
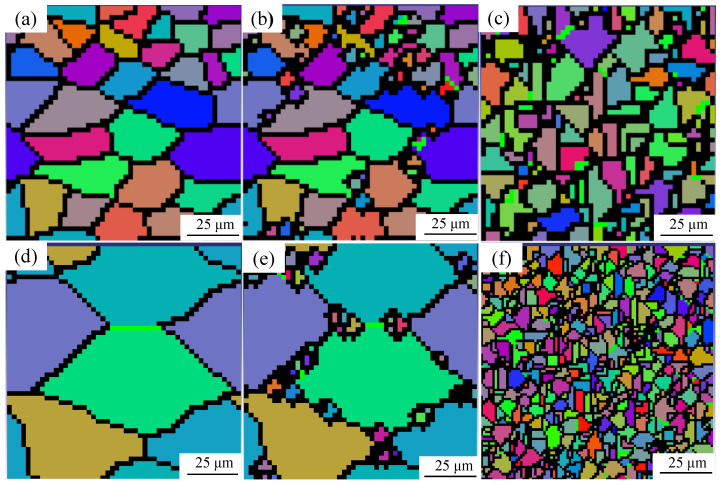
Simulated micro-structures of PH13-8Mo stainless steel (T = 900 °C, $\dot{\varepsilon }$ = 10 s^−1^): (**a**) ε = 0.3; (**b**) ε = 0.4; (**c**) ε = 0.5; (T = 1050 °C, $\dot{\varepsilon }$ = 10 s^−1^): (**d**) ε = 0.3; (**e**) ε = 0.4; (**f**) ε = 0.5.

Figure 5 shows the simulation results of the dynamic recrystallization of PH13-8Mo stainless steel at 10 s^−1^ strain and deformation temperatures of 900 °C and 1050 °C. The tissue’s grain boundaries are delineated by black lines, and the various hues represent the grains. The material’s interior zone I is hot and severely deformed, as seen in the image. Dynamic reversion occurs in the material during the initiation phase of dynamic recrystallization, causing the grains to elongate along the vector perpendicular to the compression and create fibrous stripes. The degree of dynamic recrystallization inside the material is very high when ε = 0.4, and a lot of dynamic recrystallization grains are created, which cause the recrystallization grains to grow considerably. The material has completely dynamically recrystallized, and the dynamically recrystallized grains have grown to equiaxial shape when the value of ε = 0.5. Additionally, it can be seen by comparing the deformation situation in Figure 5 that larger and nearly recrystallized grains are created as the deformation temperature rises. This is due to the fact that as the deformation temperature rises, the interatomic oscillation is boosted and the diffusion rate rises, causing a rise in grain boundary migration. This, in turn, causes a rise in the number of dynamic recrystallization nucleations and makes it possible to acquire finer grains. Additionally, it demonstrates that the impact of dynamic recrystallization grain refinement increases with increasing deformation temperature.

**Figure 6:**
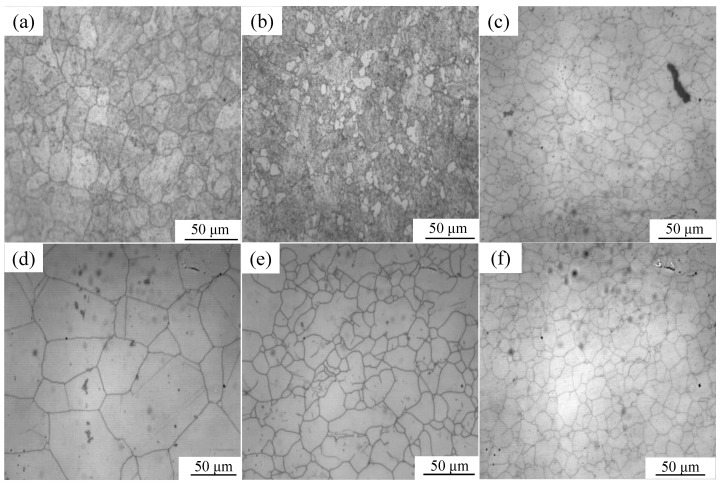
Experimental micro-structures of PH13-8Mo stainless steel (T = 900 °C, $\dot{\varepsilon }$ = 10 s^−1^): (**a**) ε = 0.3; (**b**) ε = 0.4; (**c**) ε = 0.5; (T = 1050 °C, $\dot{\varepsilon }$ = 10 s^−1^): (**d**) ε = 0.3; (**e**) ε = 0.4; (**f**) ε = 0.5.

**Figure 7:**
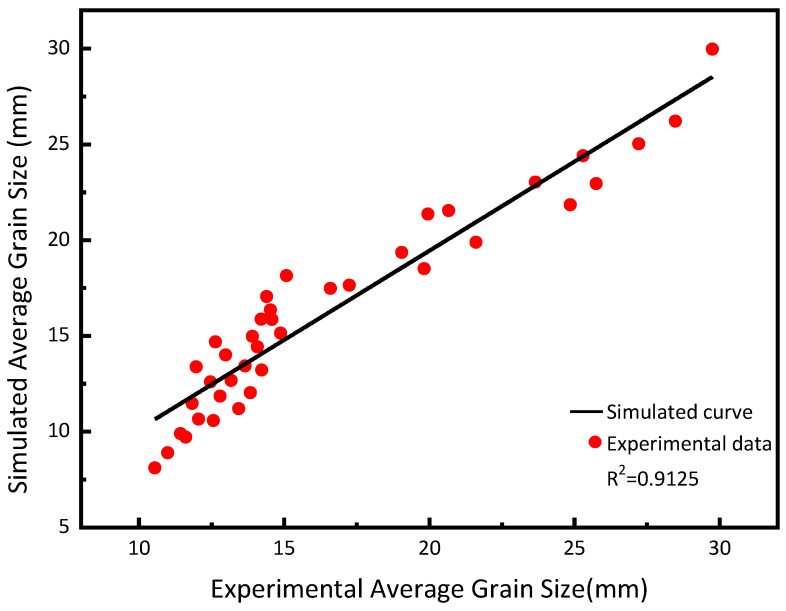
Comparison between simulated and experimental values of average grain size.

Figure 6 shows the true metallographic images of PH13-8Mo stainless steel at 10 s^−1^ strain and deformation temperatures of 900 °C and 1050 °C. When the strain reaches or exceeds the critical strain during the thermal deformation process, random nucleations occur at the grain boundaries of the original grain organization. The newly formed nuclei then migrate, grow, and consume the original grains through the grain boundaries to the surrounding area, undergoing chain-like recrystallization. This can be seen by comparing the simulation results in Figure 5 with the metallographic diagrams in Figure 6. New grain boundaries develop between the original grains and the newly recrystallized grains as a result of the simultaneous nucleation and grain growth in the tissue. The simulation results are very similar to the metallographic diagram in terms of grain distribution, grain size, and the degree of dynamic recrystallization, demonstrating the validity of this cellular automaton model. The relative error between the simulated grain size and experimental average grain size is less than or equal to 10%, with an average relative error of 6.67%, as shown in Table 2 and Figure 7; this demonstrates the model’s high degree of accuracy and the little discrepancy between simulated and experimental data. The behavior of dynamic recrystallization of PH13-8Mo alloy steel is therefore adequately explained by the constructed model, according to these data. The results of the simulation vividly and accurately reproduce the actual dynamic recrystallization process.

## 5. Conclusions

The following conclusions can be drawn from the experimental and simulation data in this work:

(1) The true stress–true strain curve of the PH13-8Mo stainless steel has obvious dynamic recrystallization characteristics under the following deformation conditions: deformation temperature of 900 °C to 1150 °C at a strain rate of 0.1~10 s^−1^. With falling deformation temperature and rising strain rate, the flow stress rises.

(2) The L–J dislocation density model for PH13-8Mo stainless steel can be expressed as follows:

$$\frac{d\rho_{i}}{d\varepsilon }=h-r\rho_{i}-rdV$$

$$r=217.41(\dot{\varepsilon }{)}^{-0.1406}exp(-\frac{79765.192}{RT})$$

$$h=1.12(\dot{\varepsilon }{)}^{0.1406}exp(\frac{79765.192}{RT})$$

(3) The L–J dislocation density model of PH13-8Mo stainless steel was imported into the DEFORM-3D micro-structure module based on the meta CA method, and the simulation results obtained were in good agreement with the actual metallographic results.

## Figures and Tables

**Table 1:** Chemical composition of PH13-8Mo stainless steel (%, mass fraction).

C	Cr	Ni	Al	Mo	N	H	Fe
0.025	12.49	8.28	1.21	0.57	0.0028	0.002	Bal.

**Table 2:** Comparison of simulated and experimental average grain sizes.

Strain Rate/s^−1^	Temperature/°C	Deformation Amount/%	Simulated Average Grain Size/µm	Experimental Average Grain Size/µm	Experimental Error/%
10	900	30	18	17	±1
		40	15	14	±1
		50	9	10	±1
	1050	30	25	24	±1
		40	21	20	±1
		50	17	18	±1

## Data Availability

The data that support the findings of this study are available from the corresponding author, upon reasonable request.
